# Evidence That the Cortical Motor Command for the Initiation of Dynamic Plantarflexion Consists of Excitation followed by Inhibition

**DOI:** 10.1371/journal.pone.0025657

**Published:** 2011-10-07

**Authors:** Wolfgang Taube, Jesper Lundbye-Jensen, Martin Schubert, Albert Gollhofer, Christian Leukel

**Affiliations:** 1 Department of Medicine, Movement and Sport Science, University of Fribourg, Fribourg, Switzerland; 2 Department of Exercise and Sport Sciences, University of Copenhagen, Copenhagen, Denmark; 3 Department of Neuroscience and Pharmacology, University of Copenhagen, Copenhagen, Denmark; 4 Spinal Cord Injury Centre, Balgrist University Hospital, University of Zurich, Zurich, Switzerland; 5 Department of Sport Science, University of Freiburg, Freiburg, Germany; Katholieke Universiteit Leuven, Belgium

## Abstract

At the onset of dynamic movements excitation of the motor cortex (M1) is spatially restricted to areas representing the involved muscles whereas adjacent areas are inhibited. The current study elucidates whether the cortical motor command for dynamic contractions is also restricted to a certain population of cortical neurons responsible for the fast corticospinal projections. Therefore, corticospinal transmission was assessed with high temporal resolution during dynamic contractions after both, magnetic stimulation over M1 and the brainstem. The high temporal resolution could be obtained by conditioning the soleus H-reflex with different interstimulus intervals by cervicomedullary stimulation (CMS-conditioning) and transcranial magnetic stimulation (TMS) of M1 (M1-conditioning). This technique provides a precise time course of facilitation and inhibition. CMS- and M1-conditioning produced an ‘early facilitation’ of the H-reflex, which occurred around 3 ms earlier with CMS-conditioning. The early facilitation is believed to be caused by activation of direct monosynaptic projections to the spinal motoneurons. CMS-conditioning resulted in a subsequent ‘late facilitation’, which is considered to reflect activity of slow-conducting and/or indirect corticospinal pathways. In contrast, M1-conditioning produced a ‘late dis-facilitation’ or even ‘late inhibition’. As the late dis-facilitation was only seen following M1- but not CMS-conditioning, it is argued that cortical activation during dynamic tasks is restricted to fast, direct corticospinal projections whereas corticomotoneurons responsible for slow and/or indirectly projecting corticospinal pathways are inhibited. The functional significance of restricting the descending cortical drive to fast corticospinal pathways may be to ensure a temporally focused motor command during the execution of dynamic movements.

## Introduction

Research investigating activity in the primary motor cortex (M1) during different phases of a finger movement in healthy subjects has indicated that at the onset of movement, unwanted contractions of adjacent muscles are prevented by inhibiting the cortical areas representing those muscles, whereas patients with focal hand dystonia show pathological overflow activation [1]. Interestingly, this spatial suppression takes place only at the onset of movement, but not during the maintenance of the contraction suggesting a specific activation of cortical neurons during the initiation of the contraction. In this study we hypothesised that if cortical activation during dynamic contractions is ‘spatially restricted’ the cortical motor command may also be restricted to certain cortical neurons within this cortical area. If so, this could relate to a preferential activation of corticomotoneurons responsible for activation of fast corticospinal projections during dynamic contractions. In 1993, Nielsen et al. introduced a conditioning method, which allows the differentiation between fast (direct) and slow(er) corticospinal pathways at rest and during activity. In those experiments, transcranial magnetic stimulation (TMS) was applied to M1 inducing a short-latency facilitation of the soleus (SOL) H-reflex, followed by a late inhibition at the onset of contraction but a late excitation during sustained contraction [2]–[4]. The authors argued that the late excitation was likely caused by activation of indirect – possibly slow conducting - corticospinal pathways and speculated that the late inhibition was due to the activation of spinal inhibitory interneurons [4]. However, they were unable to provide evidence for this hypothesis. Therefore, the current study explored the late inhibition of the conditioned soleus H-reflex further in order to highlight the involvement of different cortical neurons in the cortical motor command at the onset of dynamic contractions. For this purpose, SOL H-reflex responses of a certain size were elicited and not only cortical but also cervicomedullary stimulation was timed so that the descending corticospinal volleys coincided with the excitations generated by the Ia afferent volleys at the spinal cord. Due to the high temporal resolution of this technique (0.5 ms in this study), excitability in different fractions of corticospinal projections i.e. in the fastest, presumably monosynaptic pathways and in slower oligo- and polysynaptic pathways, could be probed and quantitatively assessed. The comparison of the results obtained after conditioning the SOL H-reflex with magnetic stimulation of the motor cortex (M1-conditioning) with the results after conditioning the H-reflex by magnetic stimulation at the cervicomedullary junction (CMS-conditioning) was used to separate cortical from spinal effects. It was hypothesized that if the observed late inhibition after M1-conditioning [3], [4] was of spinal origin, CMS-conditioning would show a similar late inhibition whereas a cortical origin would cause a late facilitation.

## Materials and Methods

### Study participants

Nine healthy subjects (age 26±4 years; 7 male and 1 female) without neurological or orthopaedic disorders participated in the present study. Eight subjects were tested during dynamic plantarflexions with both M1- and CMS-conditioning (*Protocol 1: CMS- versus M1-conditioning during dynamic plantarflexions*). In 4 subjects (three also participated in protocol 1), ISI curves during sustained isometric contractions were recorded (*Protocol 2: CMS versus M1-conditioning during sustained isometric plantarflexion*). Before testing, all subjects were informed about the experiments and gave written consent to the experimental procedure. The study was approved by the local ethics committee of the Albert-Ludwigs-University in Freiburg and experimental procedures were performed in accordance with the Declaration of Helsinki.

### EMG

EMG recordings were obtained from the SOL and tibialis anterior muscle (TA) of the right leg. After preparation, bipolar surface electrodes (Blue sensor N, Ambu®, Bad Nauheim, Germany) were attached to the skin longitudinally above the muscle belly (2 cm inter-electrode distance). The reference electrode was placed on the tibia plateau. EMG signals were amplified (×1000), bandpass-filtered (10–1000 Hz) and sampled at 4 kHz. The EMG was stored for offline analysis using custom built software (LabView® based, National Instruments®, Austin, Texas). The recordings of the TA are not displayed in the result section but were used to ensure that peripheral nerve stimulation of the tibialis nerve did not result in activation of the TA but was focused to the SOL. Furthermore, locating the hotspot for TMS of the lower leg at rest is easier for the TA in most subjects due to the lower threshold. Therefore, the TA was used as an additional/first indicator to identify the best site for stimulation. Apart from that, the recordings of the TA were also used to monitor the stimulation intensity during the M1-conditioning protocols as the threshold in most subjects is lower in TA. Consequently, the MEP served to control stimulation efficiency.

### H-reflexes

SOL H-reflexes were elicited with an electrical stimulator (constant current stimulator AS100, Alea Solutions®, Zürich, Switzerland) by stimulating the posterior tibial nerve in the popliteal fossa. Stimuli consisted of square-wave pulses of 1 ms duration. The anode, a rubber pad of 5×5 cm, was fixed on the anterior aspect of the knee just underneath the patella. The cathode (2 cm in diameter) was placed in the popliteal fossa and moved stepwise until the best position for eliciting the H-reflex was found. It was ensured that stimulation evoked no response in the TA muscle. After the optimal position was found, the cathode (Blue sensor N, Ambu®, Bad Nauheim, Germany) was fixed with tape. An H-reflex recruitment curve was obtained at rest with interstimulus intervals of 4 s while subjects were seated in the same position as during the rest of the experiment (position is described in detail later). For each subject the maximal M-response (M_max_) and the maximal H-reflex size (H_max_) were determined.

### TMS

Motor evoked potentials (MEPs) in the SOL were elicited by TMS of the contralateral motor cortical leg area (i.e. left hemisphere) using a Magstim Rapid magnetic stimulator (Magstim, Whitland, UK) with a figure-of-eight coil (90 mm Batwing). For each subject the initial stimulation point was set approximately 0.5 cm anterior to the vertex and over the midline. The handle of the coil was pointing backwards so that the first derivative induced a posterior-anterior current in the brain. The final position (hotspot) for the stimulation was determined by moving the coil anterior and left from the vertex while MEP size of SOL was monitored. The optimal position for eliciting MEPs in the SOL with minimal intensity was marked on the scalp with a felt pen. To ensure a constant position of the coil throughout the experiment, the coil was mechanically fixed. Additionally, the coil position was monitored throughout the experiment. After positioning the coil over the SOL hotspot of the cortical leg area, the subject's active motor threshold (1 AMT) was determined. AMT was defined as the intensity of magnetic stimulation required to evoke MEPs of at least 100 µV peak-to-peak amplitude in 3 of 5 consecutive trials. 1.0 AMT was expressed as a percentage of the maximum stimulator output. In both active conditions (dynamic and sustained isometric plantarflexion), the stimulation intensity was adjusted to 0.9 AMT for the conditioning trials.

### Cervicomedullary Stimulation by TMS

In each subject, cervicomedullary TMS was applied with maximum stimulator output using a Magstim® rapid magnetic stimulator (Magstim, Whitland, UK) with a double cone coil. As descending corticospinal fibres to lower leg muscles are difficult to excite at the cervicomedullar junction at rest, we used a Magstim Rapid stimulator with a biphasic pulse, because it was previously shown that for a given amplitude of initial current, biphasic stimulation was more effective than monophasic stimulation [5], [6]. The coil was positioned so that the first derivative of the induced current was cranially directed and that its centre portion set on or near the inion [7]. In both protocols, the subjects were seated in a custom built chair that fixed their legs and trunk in place and were asked to bend their back and head forward. The head rested on a custom-built table and was secured with cushions. This position was maintained throughout all experiments. In all subjects, stimulation with the maximal stimulator output (100%) was still subthreshold and therefore did not elicit detectable responses in the surface EMG of the SOL muscle. Thus, the stimulus intensity of the magnetic stimulator remained constant at its maximal output (100%) throughout the experiment. The time interval between successive stimuli was 9 sec.

### H-Reflex as a test (control) reflex

The size of the test H-reflex was measured as the peak-to-peak amplitude and was expressed as a percentage of M_max_. It has been demonstrated that the susceptibility of the H-reflex to conditioning depends on the size of the control reflex [8]. Therefore, it was ensured that the test reflex always had the same size - approximately 20% of the M_max_ - and that it was on the ascending portion of the H-reflex recruitment curve.

### M1-conditioning of the SOL H-reflex

The conditioning protocol was applied in accordance with previous studies [3], [4], [9]–[11]. Peripheral nerve stimulation at an intensity to evoke SOL H-reflexes of approximately 20% of M_max_ and TMS at an intensity of 0.9 MT were combined at different ISIs (−7, −5, −4, −3.5, −3, 0, 5, 12, 17, 22, and 27 ms). Negative interstimulus intervals (ISI) indicate that the peripheral nerve was stimulated before TMS. The latency for the TMS volley to arrive at the motoneuron is some milliseconds (∼2 to 5 ms) shorter than the arrival time of the peripheral volley. Accordingly, the earliest effect of the descending corticospinal pulse on the H-reflex can be found when the H-reflex was evoked approximately 2 to 5 ms before the TMS (ISI −5 to −2). This earliest observable H-reflex facilitation (short-latency facilitation) can (at least within the first 0.5 to 1 ms after its onset) most likely be attributed to the influence of direct monosynaptic projections from the motor cortex to spinal motoneurones of the TA and SOL muscles [3], [4]. The variation of the ISIs therefore allows differentiation between conditioning effects with respect to the descending tract (i.e. fast, supposedly monosynaptic, versus oligo- and polysynaptic corticospinal projections) [2], [3], [12], [13]. In this experiment, each ISI was measured 10 times in a randomized order. In addition, an identical number of unconditioned H-reflexes and control MEPs (without peripheral nerve stimulation) were elicited. The time interval between successive stimuli was 9 sec.

### CMS-conditioning of the SOL H-reflex

The conditioning protocol with magnetic stimulation over the brainstem resembled the “M1-conditioning” protocol described above, however, instead of stimulating the motor cortex, stimulation of the cervicomedullary junction took place with a double cone coil over the inion to condition the SOL H-reflex. As the latency of the cervicomedullary evoked volley (subthreshold “cMEP”) is a few milliseconds shorter than the latency of the cortically evoked volley (subthreshold “MEP”), different ISIs were used (−9, −8, −7.5, −7, −6, 0, 4, 12, 17, 22, and 27 ms). For each ISI, 10 measurements were obtained in a random order as well as an equivalent number of unconditioned H-reflexes and “control cMEPs” (without peripheral nerve stimulation). Due to the subthreshold nature of the transmastoid stimulation, the “control cMEP measurements” did not deviate from the baseline EMG-activity.

### Motor tasks

M1- and CMS-conditioning curves were obtained in two different motor tasks involving the triceps surae. The two tasks consisted of dynamic and sustained isometric plantarflexions, respectively. For the dynamic task, subjects were instructed to counteract as quickly as possible in response to the movement of a motor-driven footplate inducing a dynamic dorsiflexing torque (in line with reference [9]). Peak torque was adjusted to the equivalent of approximately 20–30% of the individual's maximum torque. Dynamic torque pulses were programmed as a ramp profile of 400 ms followed by a plateau of 1 s. Thus, the dorsiflexion movement was slow enough not to activate reflexive responses in the triceps surae as could be seen in an unchanged background EMG activity when subjects where told not to counteract the perturbation. In the active trials, subjects were instructed to counteract the torque as quickly as possible but should stop their contraction when they heard a tone, which occurred when the subjects reached 20–30% of their maximum torque. In previous experiments we displayed the torque level on an LED screen and subjects had to adjust their contractions to reach a predefined value as precisely as possible [9]. In the current experiment, a tone was used because subjects were seated in a bent position and could not view a monitor. The signal was considered to be important as this avoided a simple “go and give it all” contraction but forced the subjects to accurately control their contractions. Stimulation was timed so that the H-reflex and the subthreshold descending volley arrived in the muscle at the very onset of the voluntary muscular contraction. The onset of the voluntary dynamic contraction was determined in each subject after familiarization with the task in trials without stimulation and occurred around 190 ms after the beginning of the movement of the footplate (mean onset time of the voluntary contraction: 192±23 ms; mean variation of the time of onset within each subject: 15±4 ms). During sustained isometric activation, subjects had to contract with approximately 20% of their individual maximum torque and had to maintain the contraction for several minutes. From time to time, breaks of 1 to 2 minutes were given in order to avoid fatigue. To prevent any influence of fatigue, anticipation or differential levels of attention between M1- and CMS-conditioning trials, motor cortex and cervicomedullary stimulation were applied in a randomised order in a series of stimulations (interstimulus intervals of 9 seconds). Thus, subjects performed the task and did not know which kind of stimulation would occur next.

### Data analysis and statistics

**Figure 1:**
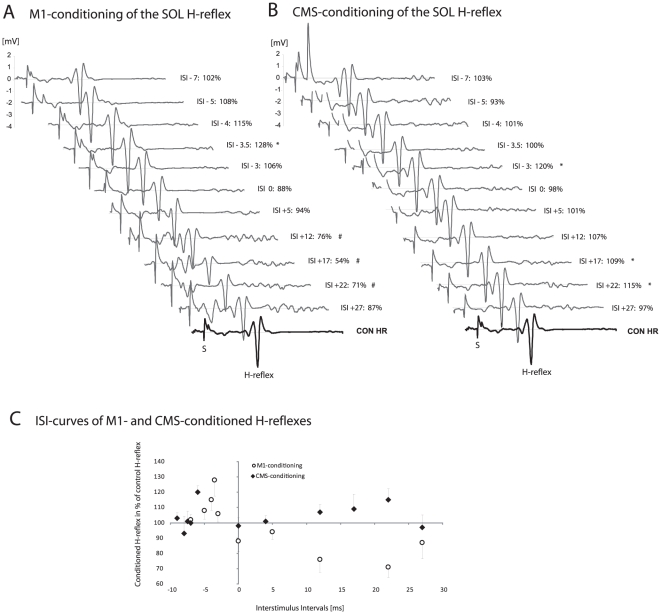
H-reflex conditioning in one representative subject. EMG traces of the soleus muscle of one single subject during conditioning of the H-reflex with cortical stimulation (M1; Figure **1A**) and brainstem stimulation (CMS; Figure **1B**) at the onset of dynamic plantarflexion. Each trace represents the mean out of 10 recorded conditioning trials. 1**A**, displayed are conditioned H-reflexes for all measured interstimulus intervals (ISIs) as well as the control (unconditioned) H-reflex (CON HR). Minus values indicate that the peripheral nerve stimulation preceded the TMS. The stimulus artefact for the electrical stimulation is marked as “S”. For every individual subject, the conditioned H-reflexes at each ISI were compared with the control H-reflexes. A significantly facilitated ISI was indicated by an * whereas a significant reduction was highlighted as #. 1**B**, conditioning effects after cervicomedullary stimulation (M1-conditioning) are illustrated for all measured ISIs in the same way as in Figure 1A (S = stimulus artefact; CON HR = control H-reflex). **1C**, to better illustrate the time course of conditioning effects, average ISI curves after M1- (○) and CMS-conditioning (⧫) are plotted in an additional graph. Each dot represents the mean out of 10 recorded conditioning trials. Fig. **1A**, **B**, and **C** display data from one and the same subject.

**Figure 2:**
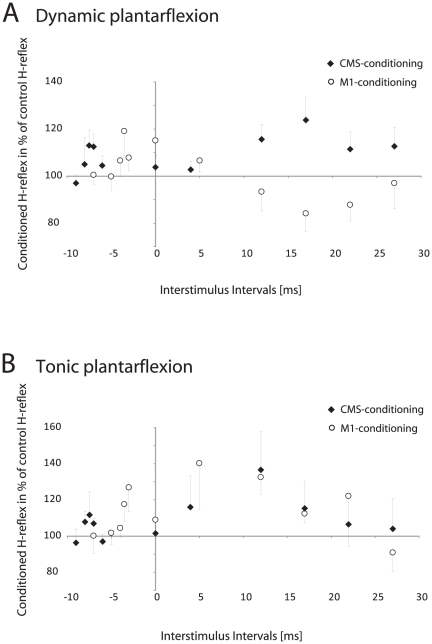
Mean data of the H-reflex conditioning. Time courses of the soleus H-reflex conditioned with magnetic stimulation of the motor cortex (○; M1-conditioning) or stimulation of the corticospinal tract (⧫; CMS-conditioning) during dynamic (**A**) and sustained isometric plantarflexion (**B**) are displayed. Dynamic plantarflexion produced an early facilitation followed by a late dis-facilitation or even inhibition (inhibition significant in 3 subjects) after M1-conditioning (data of all 8 tested subjects are displayed). In contrast, CMS-conditioning showed a late facilitation (**A**). The ISI curves after sustained isometric contraction resembled the ones obtained at rest: the early facilitation was followed by a late facilitation after both M1- and CMS-conditioning (**B**).

MEPs, unconditioned H-reflexes, conditioned H-reflexes and M-waves were expressed as peak-to-peak amplitudes in the unrectified EMG. Ten conditioned H-reflexes were averaged for each ISI with both cervicomedullary stimulation and cortical stimulation. Additionally, the 10 MEPs and 10 control (unconditioned) H-reflexes were averaged. The control H-reflexes served as a reference for the conditioned H-reflex. The intra-individual mean of the conditioned H-reflex (at each ISI) was displayed as percentage of the intra-individual mean of the unconditioned control H-reflex. Based on the intra-individual means (Fig. 1), the grand mean curve of all subjects was plotted (Fig. 2A). For every individual subject, the conditioned H-reflexes at each ISI were compared with the control H-reflexes using non-parametric Wilcoxon-tests. To statistically compare the effects of CMS- and M1-conditioning, a repeated measures ANOVA, with the factors “ISI” and “type of stimulation”, (CMS- versus M1-conditioning) [11 (ISIs) * 2 (type of stimulation)] was executed. All variables were expressed as mean ± standard error of the mean (SEM) if not indicated differently. In all figures presenting mean data, the data of all subjects who were measured in this condition were displayed. Differences were regarded significant at P<0.05 for all tests. SPSS software 16.0 (SPSS®, Chicago, Illinois) was used for the statistical analysis.

## Results

### Protocol 1: CMS- versus M1-conditioning at the onset of dynamic plantarflexions

After both CMS- and M1-conditioning, an early facilitation was observed. The occurrence of the early facilitation after M1-conditioning occurred around ISI −3.5 ms (mean ISI for the onset of facilitation −3.69±0.65 ms) while after CMS-conditioning, the early peak in facilitation was already apparent around ISI −7 ms (mean ISI for the onset of facilitation −7.19±0.59 ms). Thus, the first influence on the soleus H-reflex occurred around 3.5±0.8 ms earlier after stimulation at the cervicomedullary junction compared to stimulation over M1, which can be explained by the difference in travel distance.

TMS over M1 evoked an early facilitation followed by a late dis-facilitation or even inhibition at the onset of dynamic plantarflexion, whereas the CMS-conditioning produced both an early and a late facilitation in the mean data (type of stimulation _cMEP/MEP_: F_1,7_ = 6.19; P = 0.042; ISI * type of stimulation _cMEP/MEP_: F_10,70_ = 3.58; P = 0.001; Fig. 2A). When the ISIs were separately analyzed in each subject after M1-conditioning, 6 participants revealed a significant early facilitation and 3 of the 6 subjects displayed a significant inhibition in the ISIs ranging from +12 to +22 (Fig. 1). In all the other participants, the conditioned H-reflexes were also reduced at later ISIs but did not reach the level of significance (mean reduction of minus 11.6±21.4%). In contrast, CMS-conditioning caused significant early and late peaks of facilitation in 6 out of the 8 tested subjects (the same 6 subjects in which the early facilitation was obtained with M1-conditioning; Fig. 2A displays the data of all tested subjects (n = 8)).

Differences in the execution of the dynamic contractions during M1- and CMS-conditioning trials were extremely unlikely because all data were recorded during the same measurement and in a random order so that subjects could not anticipate which stimulus would be next. Nevertheless, to exclude the possibility of dissimilarities in the two conditions, background EMG of the soleus muscle as well as the changes in torque were assessed in a time interval 100 ms prior to the stimulation of M1- and CMS-conditioning trials. There were neither differences between the two conditions when the subjects were analysed individually (not displayed) nor when the means were compared (soleus EMG prior to M1-conditioning: 143±41 µV versus EMG prior to CMS-conditioning: 144±42 µV; P = 0.77; changes in torque in the 100 ms prior to M1-conditioning: 5±0.9 Nm versus torque changes prior to CMS-conditioning: 5±1 Nm; P = 0.47).

### Protocol 2: CMS versus M1-conditioning during sustained isometric plantarflexion

Sustained isometric contractions were evaluated in only 4 subjects as previous studies have shown that the facilitation profile after M1-conditioning resembles the profile obtained at rest, e.g. [3]. As one of these 4 subjects had a great variability in his H-reflexes he had to be excluded from analysis. The remaining 3 subjects demonstrated significant early and late facilitations with both, M1- and CMS-conditioning. As expected, no differences in the mean data between the two kinds of stimulation were evident (type of stimulation _cMEP/MEP_: F_1,2_ = 0.63; P = 0.51; ISI * type of stimulation _cMEP/MEP_: F_10,20_ = 0.67; P = 0.74; Fig. 2B).

## Discussion

The current results show that TMS over M1 elicits a short-latency facilitation followed by an inhibition/dis-facilitation of the soleus H-reflex at the onset of plantarflexion, whereas magnetic stimulation of corticospinal fibres at the level of the brainstem elicited short-latency facilitation followed by a subsequent facilitation. This suggests that the inhibition/dis-facilitation may originate from the activation of cortical neurones, which inhibit slow conducting and/or indirect corticospinal pathways to the soleus motoneurones.

### Short-latency activation of soleus motoneurones by TMS and CMS

The data obtained in relation to TMS of M1 in this study confirm the original findings by Nielsen et al. [3] and Nielsen & Petersen [2], [12]. Similar to those studies the earliest effect of TMS was a facilitation of the H-reflex at conditioning-test intervals from −3 to −5 ms, with a population average of −3.69 ms. This short-latency facilitation is in all likelihood mediated by the fastest conducting direct monosynaptic corticomotoneuronal projections to the soleus [2]–[4], [12]. The earliest effect following CMS-conditioning was also a short-latency facilitation of the soleus H-reflex. It had an average latency of around 3.5 ms shorter than the short-latency facilitation evoked by TMS, which is in all likelihood due to the shorter travel distance of this more proximal site of stimulation. As the early facilitation occurred after both kinds of stimulation, it may be reasoned that cortical neurons (corticomotoneurons) responsible for the fastest conducting corticospinal pathways contribute to the excitation seen at the onset of dynamic contractions.

### What is the origin of the late dis-facilitation/inhibition after cortical stimulation observed at the onset of dynamic contraction?

At the onset of dynamic plantarflexion three of the eight subjects showed a significant late inhibition, while the other 5 subjects demonstrated a dis-facilitation (non-significant suppression). Interestingly, CMS-conditioning instead produced a late facilitation (Fig. 2A), which was significant in 6 of the 8 tested subjects. The subjects performed the plantarflexion with the same speed and amplitude as when TMS was applied and the stimulations were elicited at the same background EMG level. Furthermore, subjects could not anticipate which kind of stimulation would follow as they were randomly applied in the same test session. Simple differences in the background excitability of the soleus motoneurones are therefore not likely to explain this profound difference between TMS and CMS.

As the motor cortex is known to have only excitatory corticospinal projections, the most straightforward explanation of the late dis-facilitation/inhibition of the H-reflex after M1-conditioning would be the activation of spinal inhibitory interneurones as also proposed by Petersen et al. [4], who observed a similar inhibition during walking. Recent evidence from the turtle spinal cord has indeed suggested that parallel excitation and inhibition of motoneurones from spinal circuitries may be a fundamental organisational principle of motor control [14]. However, this study questions that this mechanism could be responsible for the late dis-facilitation/inhibition, since a facilitation was observed following CMS-conditioning. CMS may activate the corticospinal tract in a different way compared to TMS [15] and this may provide one explanation for our observation. Another explanation, and in our opinion more likely, is that the inhibition is in fact explained by the removal of excitation of the motoneurones through slow conducting and/or indirect corticospinal pathways; i.e. that TMS may activate cortical inhibitory interneurones, which specifically inhibit the corticospinal neurones responsible for the late facilitation observed at rest and during sustained isometric plantarflexion. In this case the drop in EMG activity caused by the removal of excitation through these pathways might explain the decrease in the soleus H-reflex following TMS. CMS would as a consequence of the subcortical site of stimulation be incapable of eliciting a similar inhibitory/disfacilitatory effect. As the facilitation (CMS-conditioning) and dis-facilitation/inhibition profiles are consistent across all late ISIs, it seems that they display a universal difference between the two kinds of stimulation, which can hence most likely be attributed to the motor cortex. Theoretically, gating of input to M1 from other brain areas like for example the sensorimotor cortex (S1) could influence the pattern of dis-facilitation/inhibition [16], [17]. The late disfacilitation, seen at the onset of the movement could then be explained by a disfacilitation of the excitatory input from S1 to M1. However, previous observations point to similar gating effects in dynamic and tonic contractions [16]. Consequently, if the late disfacilitation would result from a cortico-cortical gating effect from S1 to M1, we would have expected this influence not only during ballistic but also tonic contractions. Furthermore, the onset of this gating effect is described to occur even before movement initiation [17] and it is therefore not clear how it may selectively affect cortical neurons responsible for the late and more indirect corticospinal pathways.

From a methodological point of view, the application of biphasic stimuli might have influenced the conditioning results in the present study as it was argued that mono- and biphasic stimuli activate different subsets of cortical interneurons [6]. However, comparison of the conditioning curves of the present study with previous ones [3], [4] revealed comparable effects after M1-conditioning. Furthermore, prior to the present measurements, conditioning curves obtained by mono- and biphasic stimulators were compared. Independent of the stimulator and thus the waveform the same temporal occurrence of the early and late facilitation was apparent. Thus, it seems unlikely that the stimulus-waveform notably influenced the ISI-curves of the conditioned H-reflexes in the present study.

The current observation strengthens the idea that the underlying cortical command for ballistic contractions specifically contains the activation of corticomotoneurons responsible for fast and direct corticospinal pathways. Functionally, this may allow a prompt and direct influence on the muscle activity. Consequently, this may prevent long-lasting, inflexible parts of the motor command, which might otherwise delay short-term adaptations during task execution. The inhibition of slow and indirect corticospinal pathways would also be complementary to the previously discovered ‘surround inhibition’ profile [18], which describes a suppression of cortical areas responsible for the adjacent muscles at the onset of contractions in order to avoid unwanted co-activation of those muscles [1].
